# Very low birth weight piglets show improved cognitive performance in the spatial cognitive holeboard task

**DOI:** 10.3389/fnbeh.2015.00043

**Published:** 2015-02-27

**Authors:** Alexandra Antonides, Anne C. Schoonderwoerd, Rebecca E. Nordquist, Franz Josef van der Staay

**Affiliations:** ^1^Emotion and Cognition Group, Faculty of Veterinary Medicine, Department of Farm Animal Health, University UtrechtNetherlands; ^2^Brain Center Rudolf Magnus, University UtrechtNetherlands

**Keywords:** low birth weight, pigs, spatial cognition, memory, learning, hair cortisol

## Abstract

Low birth weight (LBW) is common in humans and has been found to cause lasting cognitive and developmental deficits later in life. It is thought that the primary cause is intra-uterine growth restriction (IUGR) due to a shortage of oxygen and supply of nutrients to the fetus. Pigs appear to be a good model animal to investigate long-term cognitive effects of LBW, as LBW is common in commercially farmed breeds of pigs. Moreover, pigs are developmentally similar to humans and can be trained to perform complex tasks. In this study, we trained ten very low birth weight (vLBW) piglets and their ten normal birth weight (NBW) siblings in a spatial cognitive holeboard task in order to investigate long-term cognitive effects of LBW. In this task, four out of sixteen holes contain a hidden food reward, which allows measuring working memory (WM) (short-term memory) and reference memory (RM) (long-term memory) in parallel. Piglets were trained for 46–54 trials during the acquisition phase, followed by a 20-trial reversal phase in which a different set of four holes was baited. Both groups acquired the task and improved their performance over time. A mixed model repeated measures ANOVA revealed that vLBW piglets showed better RM performance than NBW piglets in both the acquisition and reversal phase. Additionally, WM scores in the vLBW were less disrupted than in the NBW animals when switched to the reversal phase. These findings are contrary to findings in humans. Moreover, vLBW pigs had lower hair cortisol concentrations (HCCs) than NBW pigs in flank hair at 12 weeks of age. These results could indicate that restricted intra-uterine growth causes compensatory mechanisms to arise in early development that result in beneficial effects for vLBW piglets, increasing their low survival chances in early-life competition.

## Introduction

Processes during fetal development are complex and therefore prone to disturbances and complications. Negative effects on fetal development during pregnancy can result in physical or neurological deficits and disorders later in life (Colletti, 1979). In humans, low birth weight (LBW) in babies born at term is a common phenomenon: the prevalence in developing countries ranges from 15 to 25% and is expected to be even higher as many births in such countries are not reported (Ramakrishnan, 2004). LBW in humans is defined as a weight less than 2500 g at birth and is thought to be primarily caused by intra-uterine growth restriction (IUGR) through a chronic shortage of oxygen and nutrients supply due to placental inefficiency (Biri et al., 2007; Cox and Marton, 2009). LBW has been shown to be linked to impaired cognitive function and various other deficits later in life. It is important to distinguish between LBW infants born preterm and at term (also called small for gestational age: SGA), because prematurity itself can lead to cognitive deficits (van Baar et al., 2009). SGA in humans is associated with impaired neurodevelopmental outcomes and with poorer school performance, learning difficulties and attentional problems during adolescence (Larroque et al., 2001; O’Keeffe et al., 2003; Arcangeli et al., 2012). Moreover, SGA is linked to an overall volume reduction of the brain, a decrease in white matter in both the cerebrum and cerebellum, and a small reduction of cerebellar gray matter (Martinussen et al., 2009). These cognitive deficits and behavioral problems later in life associated with SGA or LBW at term make it a pressing issue for further research. Although long-term effects of LBW have been studied in human LBW babies and children, a suitable model animal is needed to study the long-term effects of LBW on cognitive development in a more controlled manner.

In the pig industry, litter size is continuously increasing as a result of selective breeding. For example, in Denmark the average litter size increased from 11.9 piglets born alive in 2000 to 14.8 piglets in 2011 (Kondrup, 2013). The decline in birth weight of the piglets in a litter is approximately 40 g per additional piglet (Quiniou et al., 2002; Beaulieu et al., 2010). As a consequence of the larger litter sizes, the incidence of LBW piglets is increasing. Pigs can thus serve as an attractive animal model to study the effects of LBW on cognition and development. Additionally, pigs have relatively large brains and are physiologically—especially in early development—more similar to humans than for example rodents, which are more commonly used as animal models for translational research (van der Staay, 2006). Moreover, pigs are highly social and intelligent animals and can be trained to perform complex cognitive tasks (Mendl et al., 2010).

Recently, Gieling et al. (2012) studied the effects of LBW in piglets on long-term cognition, and found an indication that LBW might negatively affect cognitive development. LBW piglets had a retarded working memory (WM) performance compared to their NBW siblings at the start of the first reversal phase in a spatial cognitive holeboard task. However, these effects disappeared with further training, and no difference was found in the preceding acquisition phase. Furthermore, visual inspection of the figures in Gieling’s study suggests that RM scores for LBW animals were higher than the scores of their NBW siblings in both the acquisition and the first reversal phase. This impression, however, was not confirmed statistically. The same LBW and NBW animals were then used in a conditional discrimination task by Murphy et al. (2013). In that study, of the pigs that learned the task, the LBW animals learned the task faster than the NBW animals.

As the results from these studies are inconclusive with respect to the long-term cognitive effects of LBW in piglets, we repeated the study by Gieling et al. (2012) with stricter criteria to define LBW and tested the piglets at a younger age. Whereas Gieling et al. defined LBW as 1 SD below the average weight of a litter, we used piglet birth weight data of previous experiments to determine an upper weight limit of LBW as 1 SD below the average birth weight of nearly 500 piglets. This resulted in an upper weight limit of 1050 g which we defined as a very low birth weight (vLBW). In the current study, we examined learning and memory measures in vLBW and normal birth weight (NBW) piglets to investigate the effects of vLBW on long-term cognitive functioning. To this end, we used the spatial cognitive holeboard for pigs (Arts et al., 2009; Gieling et al., 2012). This is a free choice maze in which the animal is free to walk around and visit or revisit any site in the arena, in order to find multiple hidden rewards. By recording which sites the animal visits and revisits, working and RM can be assessed (van der Staay et al., 1990). These are forms of short- and long-term memory, respectively (Olton and Samuelson, 1976; Dudchenko, 2004).

In addition, at the end of the experiment and after euthanasia, flank hair samples from all animals were collected to determine hair cortisol concentration (HCC), which is increasingly used as a long-term biomarker for exposure to stress. Whereas cortisol concentrations in serum, saliva or urine samples are single time-point measurements which are strongly influenced by daily fluctuations, cortisol concentration in hair provides a measure for long-term or chronic stress over a prolonged time period (Russell et al., 2012). We expected that vLBW piglets would show impaired memory scores in the holeboard task compared to their NBW siblings, i.e., that they would reach lower memory scores and show longer trial durations. Additionally, we expected that vLBW animals would have higher HCCs as they face more (environmental and physical) challenges in early survival due to their developmental lag (e.g., compromised thermoregulation: Herpin et al., 2002).

## Materials and methods

### Ethics note

This study was reviewed and approved by the local ethics committee (DEC, DierExperimenten Commissie) and was conducted in accordance with the recommendations of the EU directive 86/609/EEC. All efforts were made to minimize the number of animals used and to avoid suffering.

### Subjects

Pigs ((Terra × Finnish landrace) × Duroc) born at the commercial pig breeding farm of the University Utrecht were selected. Twenty animals (ten pairs of NBW and vLBW siblings, each pair from a different litter: four pairs of female piglets, six pairs of male piglets) were selected based on their body weight measured on the day of birth. Selection occurred in two runs with 1 week in between to ensure that enough vLBW animals could be selected for the study. The vLBW animals were selected based on two criteria: a minimum of 1 SD below the average birth weight of the study population (based on the birth weights of 484 piglets; this yielded at cut-off of <1050 g) and from a litter containing a minimum of 10 piglets. The NBW piglets were selected based on the average birth weight of the litter with the same sex as the selected vLBW piglet. Of all piglets, head size (snout to the back of the cranium) and total body length (snout to tail base) were measured on the day of birth to check for asymmetrical growth as an additional measure for intra-uterine growth retardation (IUGR; Amdi et al., 2013). In order to increase survival rates of the vLBW piglets during the first days, close monitoring and hand feeding of sow milk (once per day) were applied. NBW siblings were handled for the same amount of time. One male piglet of the vLBW group was euthanized due to lasting illness during the habituation period, thus the experiment was conducted with 19 piglets in total.

### Housing

Selected piglets were transported to our research facility at age 4–7 days and were housed by birth weight class and age in groups of five animals each in four adjacent pens (1.25 × 2.50 m until 10 weeks of age, then until 12 weeks of age 2.50 × 2.50 m) containing sawdust bedding, straw and toys. Temperature was gradually decreased from 26°C in the first weeks to 21°C at the end of the study. During the first week, a heat lamp was hung 1 m above each pen. A 12/12 h light/dark cycle was applied with lights on at 7 a.m. A radio played continuously; slightly louder at daytime (7 a.m. to 4 p.m.) than at night. Water was provided ad libitum. Pigs were fed milk replacer (Milkiwean Yoghurt, Trouw Nutrition, Nutreco Global, The Netherlands) for the first 4 weeks. In addition to the milk replacer, commercial piglet feed was provided. Piglets were gradually weaned between three and four weeks of age, after which they were fed a balanced commercial pig feed.

### Apparatus

**Figure 1:**
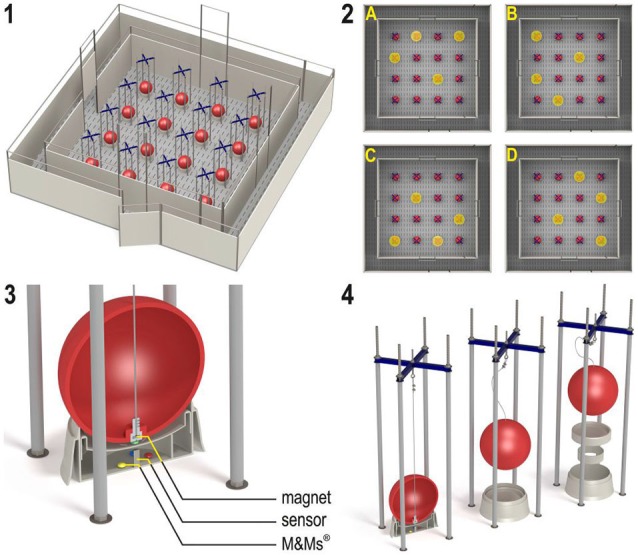
**(1)** The spatial cognitive holeboard for pigs. **(2)** The four patterns of baited holes. **(3), (4)** Constructional details of the holes. Each hole—a fool bowl with a false bottom under which three M&M’s® chocolates are placed in order to mask odor cues—is covered by a red ball. Each food bowl is equipped with a sensor that sends a signal to the computer if the contact with the magnet in the ball is interrupted; i.e., when the pig lifts the ball with its snout (illustrations: Yorrit van der Staay).

The holeboard apparatus (Ossendrijver BV, Achterveld, The Netherlands) consisted of a 360 × 360 cm square arena with a 4 × 4 matrix of food bowls, surrounded by a small corridor (40 cm) with a slatted black synthetic floor (Figure 1, panel 1). The synthetic walls were 80 cm high and had a steel bar on top (total height: 1 m). The apparatus was elevated 25 cm off the floor. The arena could be entered through four different guillotine doors, one on each side, which were operated from the outside using a rope and pulley system. Piglets entered the holeboard through the main entrance and always turned left into the corridor until they found an open door, through which they entered the testing arena. Piglets inside the holeboard arena were able to see the surrounding walls of the experimental room and the ceiling with two rows of fluorescent tubes, as well as the two experimenters standing in front of the holeboard directly right of the main entrance door. The experimenters avoided eye contact with the piglets during trials. Auditory extra-maze cues were a radio that was playing continuously, and the piglet’s pen mates in the waiting area in front of the holeboard apparatus, where they were housed during testing.

The food bowls were covered by red plastic balls which could be lifted by the piglets with their snout (JollyBall Dog Toy, ø 24 cm, 400 g), to prevent the piglets from finding the rewards by sight. To ensure that the rewards were not found by smell, every food bowl contained three rewards (replaced daily) in a false bottom (Figure 1, panel 3). The apparatus was cleaned at the end of each testing day and after a trial if an animal had defecated during a trial. Hole visits were automatically recorded using custom made software (Blinq Systems, Delft, The Netherlands). A visit was scored when a pig lifted the ball and the connection between the magnet in the ball and the sensor in the food bowl was broken (Figure 1, panel 4). This signal was registered by an interface (LabJack) and sent to a PC. If the same ball was lifted again within 10 s and no other hole was visited in between, this was not counted as a revisit. A trial started when a pig entered the arena with both front legs and ended when a piglet found all four rewards or when the maximum time of 450 s was reached (whichever event occurred first), after which the piglet was allowed to leave the arena through the door closest to the main entrance door.

### Training and testing

During the first 3 weeks after arrival into the new pens, all piglets were gradually habituated to the two experimenters, the hallway leading to the holeboard and the holeboard itself, in sessions of 10–30 min per day. Training occurred with mini marshmallows as reward, as these were easy to consume for the young animals. In the testing phase, M&M’s® chocolates were used as reward. Holeboard testing started when all piglets had learned to lift balls with their snout in order to find rewards and were comfortable to enter the arena alone, which was at approximately 7 weeks of age. Before testing, six habituation trials (two trials per day, 3 days in total) were conducted in which all 16 holes were baited with a food reward. Then, each animal was assigned its own rewarded configuration, in which 4 of the 16 holes were baited. In total, four different configurations were used, in such a way that every hole was baited equally often (Figure 1, panel 2). All piglets received two trials in close succession per day on the first four testing days (total: eight trials), after which they were tested in four massed trials per day. The entrance door was randomly assigned per trial by the software. After a predetermined learning criterion was reached (average RM score > 0.6 over the last four trials), which was after at least 46 acquisition trials and at most 54 trials, the animals moved to the reversal configuration. The reversal configuration was the mirror image of the configuration used during the acquisition phase (A to C, B to D, C to A, B to D; Figure 1, panel 2). All piglets received 20 reversal trials, thus in total all piglets received at least 74 trials. At the end of the experiment (at 12 weeks of age), all animals were euthanized by an intracardial injection with an overdose of pentobarbital (Euthasol®, AST Farma B.V. Oudewater, The Netherlands), after which brains were dissected and weighed.

### Hair samples

At the end of the experiment and after euthanasia, hair samples (0.5–1 g) were taken from the left flank of each animal with a trimmer. Of each hair sample, 250 mg was washed, dried and ground with a bead beater for 30 min in steel micro vials containing three 1 mm steal beads. Thereafter, 50 mg of each powdered sample was collected in a micro-centrifuge tube. 1 ml methanol was added after which the samples were incubated at room temperature for 24 h with slow rotation to extract steroids. Of the extract, 0.6 ml was placed in a new tube and dried at 45°C in a heating block overnight. The dried extracts were dissolved in 0.4 ml phosphate buffer. Cortisol concentrations were then determined in duplo using a Salivary Cortisol ELISA kit (Salimetrics LLC, PA, USA). As one vLBW animal was euthanized at 4 weeks of age due to lasting illness, the hair of another vLBW animal was unusable and the ground hair sample of one NBW animal was lost accidentally, cortisol concentration in hair samples of 8 vLBW and 9 NBW pigs was determined.

### Statistics

From the holeboard data the following measures were calculated after either all rewards were found or the maximum time of 450 s had elapsed, whichever event occurred first (van der Staay et al., 2012): (1) Reference memory (RM), a ratio that is defined by the number of visits and re-visits to the rewarded set of holes divided by the number of visits and re-visits to all holes; (2) Working memory (WM), a ratio defined by the number of visits that yield a food reward divided by the number of visits and re-visits to the rewarded set of holes; (3) Trial duration (TD), the time between entering the holeboard and finding all four rewards (when not all rewards were found the maximum trial duration of 450 s was recorded); (4) Inter-visit interval (IVI), the average time between two hole visits; (5) Latency to the first visit (LFV); (6) Total visits (TV), unrewarded visits (URV) and rewarded visits (RV); and (7) Number of visits until 1st (Vfirst), 2nd (Vsecond), 3rd (Vthird) and 4th (Vfourth) reward found (Gieling, 2013). These variables include measures for both memory performance (RM, WM) and for motivation or speed (TD, IVI).

The trials of the actual holeboard testing were analyzed using the mean of four trials resulting in trial blocks, except for the first block, which was the mean of six trials. Of all animals, the first 46 acquisition trials thus divided into 11 trial blocks (block 1–11) were analyzed, yet not the extra acquisition trials that a piglet received when it had not yet reached the criterion of RM > 0.6 after 46 trials. The following 20 reversal trials were also analyzed in blocks of 4 trials, thus divided into 5 trial blocks (block 12–16). The holeboard data analyses were performed for three different phases: acquisition, transition and reversal. The transition phase is the switch from the acquisition phase to the reversal phase, i.e., the last trial block of the acquisition compared to the first trial block of the reversal (block 11 compared to block 12). This is a measure of the response flexibility of an animal: a large difference means that the animal faced difficulties to adapt to the new situation.

All analyses were performed using the statistical software SAS (version 9.4, SAS Institute, Cary, NC, USA). Normal distribution of all variables was assessed using the Shapiro-Wilk test (SAS PROC UNIVARIATE). All variables expressing latencies or durations were log10-transformed to meet the normality assumption.

The effects of birth weight on the growth curves were analyzed with a mixed model analysis of variance (ANOVA) to account for clustering of piglets within litters and repeated measurements within piglets, with the fixed effects Birth weight (vLBW vs. NBW), Week, and Birth weight*Week.

Effects of birth weight on the habituation to the holeboard (six successive trials), on the learning curves of the acquisition phase (11 successive trial blocks) and reversal phase (5 successive trial blocks), and on the transition between the acquisition and reversal phase were analyzed using mixed model ANOVAs. For holeboard habituation, fixed effects were Birth weight, Trials (six successive trials) and the Birth weight*Trials interaction. For holeboard acquisition, transition and reversal, fixed effects were Birth weight, Trial blocks and Birth weight *Trial blocks.

The effects of birth weight on cortisol, head length in cm, full body length in cm, and head length as percent of full body length were analyzed using a mixed model ANOVA with the fixed effect Birth weight. In all mixed model analyses, a random effect for litter was added, and the correlation of repeated measures within piglets was addressed using an autoregressive (1) structure for the residuals (SAS PROC MIXED).

## Results

### Cognitive holeboard performance

Table 1 shows the results of statistical analyses for all measures. As RM and WM are the most important measures of memory performance and TD and IVI the most important motivational measures, these four variables will be discussed in more detail.

**Table 1:** **Performance of vLBW and NBW piglets in the spatial cognitive holeboard task during habituation (Hab), and during the acquisition (Acq), transition (Trans), and reversal (Rev) phase**.

	Holeboard habituation									
		Birth weight			Trials			Birth weight × Trials		
Measure	Phase	*F*	df	*P≤*	*F*	Df	*P≤*	*F*	df	*P≤*
**Total number of visits (TV)**	Hab	0.42	1,88	0.5201	1.60	5,88	0.1680	0.48	5,88	0.7915
**Number of rewards found (REW)**	Hab	0.07	1,88	0.7860	2.04	5,88	0.0810	0.47	5,88	07955
	**Holeboard acquisition (Acq), transition (Trans), reversal (Rev)**									
		**Birth weight**			**Trial blocks**			**Birth weight × Trial blocks**		
**Measure**	**Phase**	***F***	***df***	***P≤***	***F***	**Df**	***P≤***	***F***	**df**	***P≤***
**Working memory (WM)**	Acq	0.02	1,178	0.8845	6.94	10,178	**<0.0001**	1.83	10,178	0.0589
	Trans	31.91	1,25	**<0.0001**	44.15	1,25	**<0.0001**	2.61	1,25	0.1190
	Rev	0.80	1,76	0.3746	2.37	4,76	**<0.0001**	2.37	4,76	0.0596
**Reference memory (RM)**	Acq	25.11	1,178	**<0.0001**	41.26	10,178	**<0.0001**	5.02	10,178	**<0.0001**
	Trans	11.06	1,25	**0.0027**	130.35	1,25	**<0.0001**	8.92	1,25	**0.0062**
	Rev	18.54	1,76	**<0.0001**	50.47	4,76	**<0.0001**	6.67	4,76	**0.0001**
**Trial duration (TD)**	Acq	0.33	1,178	0.5649	10.79	10,178	**<0.0001**	1.89	10,178	**0.0485**
	Trans	1.44	1,25	0.2419	114.69	1,25	**<0.0001**	0.00	1,25	0.9894
	Rev	0.37	1,76	0.5464	26.68	4,76	**<0.0001**	0.27	4,76	0.8945
**Latency first visit (LFV)**	Acq	2.56	1,178	0.1114	1.74	10,178	0.0756	0.63	10,178	0.7858
	Trans	0.40	1,25	0.5319	0.03	1,25	0.8576	0.00	1,25	0.9876
	Rev	1.96	1,76	0.1654	1.65	4,76	0.1702	0.73	4,76	0.5716
**Inter-visit-interval (IVI)**	Acq	1.90	1,178	0.1696	2.96	10,178	**0.0018**	0.86	10,178	0.5687
	Trans	0.01	1,25	0.9172	7.96	1,25	**0.0092**	0.35	1,25	0.5579
	Rev	0.23	1,76	0.6324	3.20	4,76	**0.0174**	0.15	4,76	0.9638
**Total number of visits (TV)**	Acq	14.36	1,178	**0.0002**	21.78	10,178	**<0.0001**	1.40	10,178	0.1818
	Trans	9.89	1,25	**0.0043**	113.18	1,25	**<0.0001**	0.98	1,25	0.3314
	Rev	9.06	1,76	**0.0035**	24.60	4,76	**<0.0001**	0.89	4,76	0.4747
**Unrewarded visits (URV)**	Acq	17.97	1,178	**<0.0001**	24.64	10,178	**<0.0001**	1.29	10,178	0.2411
	Trans	8.52	1,25	**0.0073**	131.10	1,25	**<0.0001**	0.45	1,25	0.5068
	Rev	11.24	1,76	**0.0012**	29.36	4,76	**<0.0001**	0.82	4,76	0.5150
**Rewarded visits (RV)**	Acq	0.85	1,178	0.3578	6.27	10,178	**<0.0001**	1.81	10,178	0.0613
	Trans	12.25	1,25	**0.0018**	17.19	1,25	**0.0003**	3.70	1,25	0.0657
	Rev	1.24	1,76	0.2686	6.67	4,76	**0.0001**	2.16	4,76	0.0811
Visits before 1st reward (Vfirst)*	Acq	2.74	1,178	0.0996	5.24	10,178	**<0.0001**	0.98	10,178	0.4617
	Trans	0.10	1,25	0.7555	35.26	1,25	**<0.0001**	0.77	1,25	0.3873
	Rev	3.71	1,76	0.0579	5.73	4,76	**0.0004**	1.44	4,76	0.2286
Visits before 2nd reward (Vsecond)*	Acq	8.24	1,178	**0.0046**	12.62	10,178	**<0.0001**	1.81	10,178	0.0621
	Trans	0.06	1,25	0.8072	61.75	1,25	**<0.0001**	0.67	1,25	0.4203
	Rev	2.27	1,76	0.1360	16.24	4,76	**<0.0001**	1.79	4,76	0.1400
Visits before 3rd reward (Vthird)*	Acq	21.12	1,178	**<0.0001**	19.63	10,178	**<0.0001**	1.40	10,178	0.1822
	Trans	1.68	1,25	0.2062	85.36	1,25	**<0.0001**	0.19	1,25	0.6701
	Rev	18.78	1,76	**<0.0001**	23.61	4,76	**<0.0001**	1.03	4,76	0.3963
Visits before 4th reward (Vfourth)*	Acq	10.16	1,178	**0.0017**	23.65	10,178	**<0.0001**	1.22	10,178	0.2827
	Trans	3.40	1,24	0.0776	86.08	1,24	**<0.0001**	0.03	1,24	0.8634
	Rev	7.17	1,73	**0.0092**	19.36	4,73	**<0.0001**	0.27	4,73	0.8969

*For further information about the operational definitions of these variables, see Gieling et al. (2014).

#### Habituation trials

Birth weight did not affect habituation to the holeboard apparatus. Both birth weight groups had a similar Total number of hole visits (TV) and found a similar number of rewards (REW).

#### Working memory

**Figure 2:**
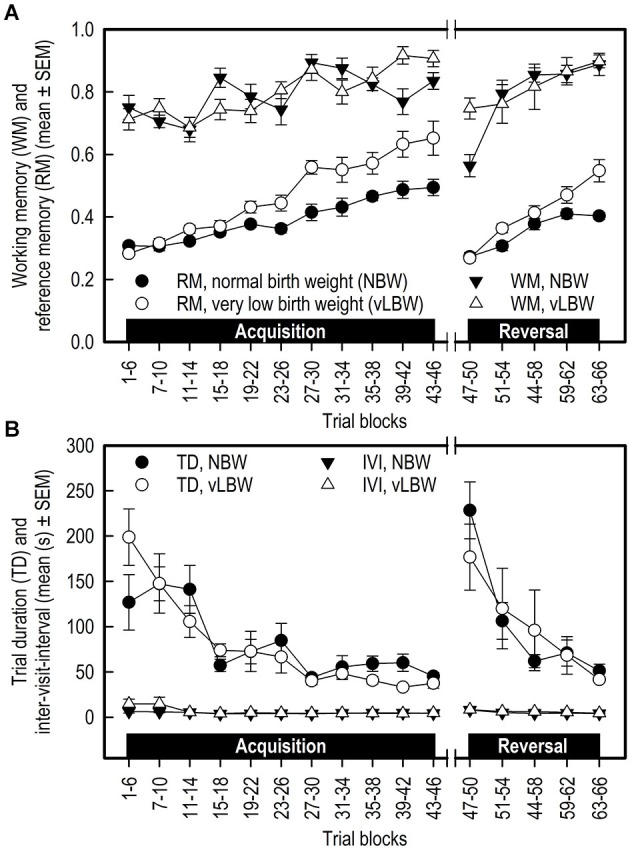
**(A)** Working memory (WM) and reference memory (RM) performance and **(B)** Trial duration (TD) and inter-visit interval (IVI) of NBW (*n* = 10) and vLBW (*n* = 9) piglets in the spatial cognitive holeboard task during the acquisition phase (trials 1–46) and the reversal phase (trials 47–66). Note that TD and IVI were analyzed statistically after log_10_ transformation whereas the untransformed means and SEMs are depicted here.

Acquisition: Working memory (WM) performance increased for both birth weight groups during the acquisition phase. WM performance did not differ between the two birth weight groups (Birth weight: *F*_(1,178)_ = 0.02; *p* = 0.8845; see Figure 2A; Table 1). There was a strong trend of an interaction between Birth weight and Trial blocks on WM performance in the acquisition phase (*F*_(10,178)_ = 1.83; *p* = 0.0589). However, further analysis of the data revealed that this interaction was due to a difference in performance between the birth weight groups in trials 15–18 (*F*_(1,262)_ = 3.83; *p* = 0.0515) and trials 39–42 (*F*_(1,262)_ = 7.25; *p* = 0.0076), thus not due to a systematic difference in performance between NBW and vLBW piglets.

Transition: The WM performance dropped to a lower level when the piglets were presented a new set of baited holes at the start of the reversal phase (Trial blocks: *F*_(1,25)_ = 44.15; *p* < 0.0001). The WM performance of the NBW piglets was lower than that of the vLBW piglets (Birth weight: *F*_(1,25)_ = 31.91; *p* = < 0.0001). Although visual inspection of Figure 2A suggests that the drop in performance was stronger in the NBW piglets, this impression was not confirmed statistically (Birth weight by Trial blocks interaction: *F*_(1,25)_ = 2.61; *p* = 0.1190).

Reversal: After the drop in performance caused by presenting a new pattern of baited holes, the WM performance increased again during the reversal phase (Trial blocks: *F*_(4,76)_ = 2.37; *p* < 0.0001). A strong trend of an interaction effect between Birth weight and Trial blocks on WM in the reversal phase (*F*_(4,76)_ = 2.37; *p* = 0.0596) was found, suggesting that the improvement in WM performance of the NBW piglets was stronger than that of the vLBW piglets during reversal training.

#### Reference memory

Acquisition: The vLBW piglets had, on average, higher RM scores than the NBW piglets (Birth weight: *F*_(1,178)_ = 25.11; *p* < 0.0001; see Figure 2A; Table 1). The vLBW piglets learned the RM component of the holeboard task faster than their NBW littermates (Birth weight by Trial blocks interaction: *F*_(10,178)_ = 5.02; *p* < 0.0001) and reached a higher performance level at the end of the acquisition phase.

Transition: Reference memory performance decreased to chance level in both groups (Trial blocks: *F*_(1,25)_ = 130.35; *p* < 0.0001). The decrease was larger in the vLBW piglets than the NBW piglets (Birth weight by Trial blocks interaction: *F*_(1,25)_ = 8.92; *p* = 0.0062).

Reversal: Both groups improved RM performance across the 5 trial blocks of the reversal phase (Trial blocks: *F*_(4,76)_ = 50.47; *p* = 0.0001), but improvement of the vLBW piglets was faster than that of their NBW littermates (Birth weight by Trial blocks interaction: *F*_(4,76)_ = 6.67; *p* < 0.0001).

The NBW group had a larger total number of hole visits (TV) and URV than the vLBW group in all phases, which is in line with the finding that birth weight affected RM performance in all three phases (Table 1).

#### Trial duration

Acquisition: In both groups, the trial duration decreased over time (Trial Blocks: *F*_(10,178)_ = 10.79; *p* < 0.0001; Figure 2B; Table 1), but this decrease was stronger in the vLBW piglets (Births weight by Trial blocks interactions: *F*_(10,178)_ = 1.89; *p* = 0.0485), presumably because the vLBW piglets had longer trial durations during the first block of acquisition.

Transition: The trial duration increased from the last acquisition to the first reversal trial block (Trial Blocks: *F*_(1,25)_ = 114.69; *p* < 0.0001). This increase was not affected by birth weight (Birth weight: *F*_(1,25)_ = 1.44, *p* = 0.2419; Birth weight by Trial blocks interaction: *F*_(1,25)_ = 0.00; *p* = 0.9894).

Reversal: The trial duration decreased similarly in both groups of piglets (Table 1).

#### Inter-visit interval

Acquisition: The time needed per hole visit, i.e., the inter-visit interval (IVI) decreased similarly in both groups across the 11 successive acquisition trial blocks (see Figure 2B; Table 1).

Transition: Introducing a new pattern of baited holes in the reversal phase increased the IVI. The increase was not affected by birth weight.

Reversal: The IVI during the reversal phase decreased slightly across the five successive reversal trial blocks. The decrease was unaffected by birth weight. IVI is a measure of how fast the animal searches for rewards, and may thus provide an indication of how motivated the animal is to complete the task.

### Growth

**Figure 3:**
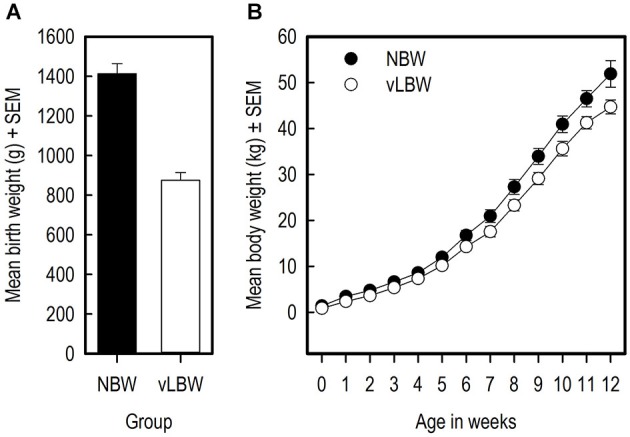
**Weights and growth of the piglets. (A)** The birth weights of the vLBW and NBW piglets in grams. **(B)** The body weight of the piglets in kilograms over the course of the experiment.

The NBW piglets had on average a higher birth weight than the vLBW piglets (Figure 3A; *t*_(9)_ = −10.70; *p* < 0.0001). Over the course of the experiment, the weight of the NBW group remained higher than that of the vLBW group (*F*_(1,216)_ = 84.04; *p* = < 0.0001; see Figure 3B). Moreover, the vLBW piglets had a slower growth rate than the NBW piglets (*F*_(12,216)_ = 2.57; *p* = 0.0033). The head size relative to the full body length on the day of birth did not differ between the groups (*t*_(8)_ = 0.28; *p* = 0.782).

### Brain weights

The relative brain weight was calculated by dividing the brain weight by the total body weight at the end of the experiment, when the piglets were 12 weeks old. There was a strong trend to higher absolute brain weights in the NBW group compared to the vLBW group (*t*_(8)_ = −2.30; *p* = 0.050). The relative brain weights did not differ between the groups (*t*_(8)_ = 1.26; *p* = 0.241).

### Hair cortisol concentrations

**Figure 4:**
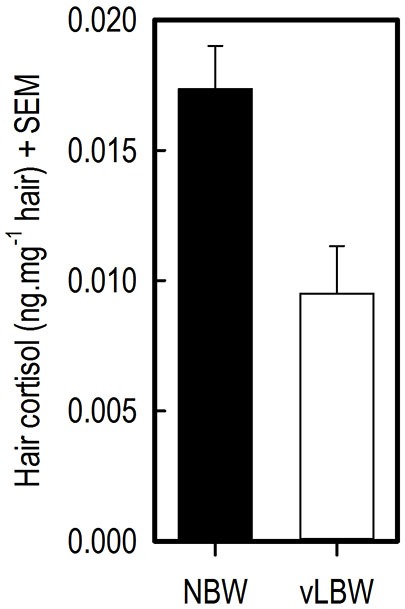
**Hair cortisol concentrations (HCCs) in flank hair of 12-week-old NBW (*n* = 9) and vLBW (*n* = 8) piglets**.

Cortisol concentration in flank hair from the vLBW pigs at 12 weeks of age was lower than that of NBW pigs (*t*_(7)_ = −4.60; *p* = 0.0025; see Figure 4).

## Discussion

The aim of this study was to investigate long-term cognitive performance of vLBW piglets compared to their NBW siblings. As human studies show that LBW children born at term show (neuro) developmental impairments later in life (e.g., O’Keeffe et al., 2003), we expected to find deficits in learning and memory performance in vLBW piglets.

Our results confirm previous findings that young piglets are able to acquire the holeboard task. Memory scores improved and latencies declined over the course of the experiment for all piglets. Although we attempted to test the piglets in our study at a younger age than previous studies using the holeboard task for pigs (Arts et al., 2009 (9 weeks); Haagensen et al., 2013 (6–7 weeks, minipigs); Gieling et al., 2014 (7–8 weeks)), the piglets were not able to perform the task until they were approximately 7 weeks of age. It can therefore be assumed that a certain level of physical and mental development needs to be achieved in order to perform the holeboard task, which the piglets reach at about 7 weeks of age. Moreover, it appears that the animals are not comfortable to be alone in the holeboard arena before that age.

The body weights of the vLBW group remained lower than the NBW group throughout the experiment. Thus, vLBW piglets did not show compensatory growth, which is in line with previous studies of effects of birth weight on growth performance in pigs (Gondret et al., 2005; Rehfeldt and Kuhn, 2006).

### Improved cognitive performance

Performance in the holeboard task was opposite to what we expected and differs from findings in human studies. In the current study, vLBW piglets showed a faster acquisition and reached a higher performance level in the holeboard task than the NBW animals. Previous studies conducted by Gieling et al. (2012) and Murphy et al. (2013) on the cognitive effects of LBW in pigs used less strict criteria when selecting LBW pigs, and their results on the effects of LBW on long-term cognition were inconclusive. In the present study, stricter selection criteria for LBW animals were applied—the most prominent being a lower birth weight limit as criterion for vLBW—which may explain why results were supported statistically in the present study, whereas they were not in previous studies on cognitive effects of LBW in pigs. The average weight difference between the LBW and NBW pigs was 549 g in the study of Gieling et al., whereas it was 539 g in our study. However, when comparing the birth weights of all piglets of that study to the current study, there is a 631 g difference in average birth weight between piglets used. This results in lower absolute birth weights of the piglets selected for the current study and thus more extreme LBWs in our vLBW group than in the LBW group of Gieling et al., which may explain the differences in our findings.

Although we fed the vLBW additional sow milk by hand feeding them in the first days after birth, we do not expect our findings on cognitive performance to be due to this difference. Hand feeding these vLBW animals was often unsuccessful as the animals struggled and did not ingest much of the milk they were offered, which was once a day in the first 4 days. We furthermore expect that NBW animals in general ingest more sow milk due to their stronger chances in teat competition. All animals were removed from the sow after 4–6 days, after which all animals received the same amount of milk replacer. Thus, we do not expect that the small amount of additional feeding to the vLBW animals had any effects on cognitive results in the holeboard test.

LBW pigs may have developed mechanisms to compete with their larger siblings in order to increase their low survival chances. It is possible that growth restriction due to (mild) intra-uterine hypoxia or ischemia in vLBW pigs causes a process called brain preconditioning to occur, in which post- or early prenatal sublethal stressors induce protection against other future stressors or injuries (for a review, see Stetler et al., 2014). Although this is mere speculation, the results of the current study do indicate that the occurrence of LBW in pigs probably involves or triggers other mechanisms than LBW in humans, as our results are opposite to findings in human LBW or SGA infants. Such compensatory mechanisms as brain preconditioning resulting from intra-uterine stress could make the animal able to cope with stress and competition better, which would be advantageous for LBW pigs competing for resources in large litters. Humans do not have this early postnatal competition, as they are usually born with only one or two babies at a time. Thus, the difference in effects of LBW on long-term cognition between humans and pigs may be due to the difference in early-life competition for resources. Another possible explanation for the fact that LBW animals performed better may be that they are more strongly motivated to obtain a food reward than the NBW animals. Measures in the holeboard task that can provide an estimation of the motivation of the animals are the latency of the first visit and inter-visit interval (van der Staay et al., 2012). The birth weight groups did not, however, differ for these measures. It may still be interesting to further investigate motivation for food rewards in LBW and NBW pigs in future studies, using more specific tests designed to measure motivation.

Previous studies defined LBW in piglets as 2 SD (Cooper, 1975) or 2.5 SD (Gondret et al., 2005) below the average weight, whereas we used 1 SD below the average weight of the study population. These stricter criteria, however, do require more intensive postnatal care of the piglets as survival chances are low in piglets with vLBW. Reduced vigor and thermoregulation are amongst the main causes of neonatal mortality in vLBW piglets (Tuchscherer et al., 2000; Herpin et al., 2002). Thus, extra care is needed in order to increase survival chances of vLBW piglets. In order to provide the required extra care and allow close monitoring of vLBW piglets, intensive care units similar to those used for human neonates have been developed (e.g., Lennon et al., 2011). In the set-up of the current study, the available infrastructure could not provide this extra care. It may be useful to use stricter criteria for LBW animals in future studies and raise these animals under intensive care conditions.

### Growth retardation and (a)symmetry

The total body length and head size measured on the day of birth were used to check for asymmetrical growth as an additional measure for IUGR. Asymmetrical IUGR is the most common form of IUGR in humans (70%) and is a sign of head or brain sparing in the third trimester of pregnancy, whereas symmetrical IUGR is thought to find its onset much earlier in the course of pregnancy (Lin et al., 1991). Severe IUGR piglets have been shown to have higher relative brain weights than mild IUGR piglets, and these in turn have larger relative brain weights than NBW piglets (Amdi et al., 2013). In the current study, a strong trend to larger absolute brain weights in the NBW piglets than the vLBW piglets was found. This is a consequence of the larger total size of the NBW piglets. However, the relative size of the head did not differ between the vLBW and NBW groups on the day of birth, ruling out that asymmetrical growth has occurred in the vLBW animals. This is an indication that growth retardation in our vLBW piglets had an early onset in the course of pregnancy and brain sparing did not occur in these animals, i.e., that the present vLBW piglets are not modeling IUGR. In a study investigating indicators of neonatal survival in piglets, birth weight was shown to be a critical factor with respect to mortality in live-born piglets (Baxter et al., 2008). However, as regards mortality in still-born piglets, shape and size of the piglets (as measured by ponderal index and body mass index) appeared to be better indicators for survival. Piglets showing asymmetrical IUGR thus have a high prevalence of prenatal mortality. This might explain why we did not find any asymmetrical growth in the vLBW piglets that were available for selection in our study.

### Hair cortisol concentrations

Significantly lower HCC in flank hair of vLBW than NBW piglets at 12 weeks of age were found. This implies that the vLBW animals experienced less stress over the course of their lives. For example, in humans, traumatized patients with PTSD had higher HCC than controls without PTSD symptoms, and in both groups the number of traumatic life events positively correlated with HCC (Steudte et al., 2011). In dogs, salivary cortisol concentrations measured in their home environment positively correlated with HCC (Bennett and Hayssen, 2010). Similarly, HCC in rhesus macaques correlated with saliva samples taken from animals that were trained for saliva collection (Davenport et al., 2006). These studies show that hair is a reliable medium for measuring basal cortisol concentrations.

The difference in HCC between vLBW and NBW pigs may be due to the difference in performance between the two groups, as making more mistakes in the task and thus performing worse may have caused slightly more distress in the NBW animals over time than in the vLBW animals. As the animals were tested in the holeboard task from age 7–12 weeks, after which the hair was collected, stress levels are likely to be at least partially influenced by the effects of holeboard testing. Another plausible explanation is that the NBW animals experienced more stress due to less space per pig they had available in their home pen. Home pens measured the same for all groups of five animals (1.25 × 2.50 m until 10 weeks of age, then until 12 weeks of age 2.50 × 2.50 m); while the NBW animals were significantly heavier—and thus larger—than the LBW pigs during the entire course of the experiment. Moreover, one LBW animal was euthanized at 4 weeks of age, thus one LBW group was housed with four pigs in their home pen. This reduced space per pig in the home pen for NBW animals may have caused elevated stress levels in the NBW pigs as compared to the LBW animals. In future studies comparing stress levels in pigs, housing and space per pig should thus be taken into account.

An alternative explanation is that vLBW pigs are somehow less affected by stressors than NBW pigs. Human small for gestational age (SGA) infants show a blunted stress response to a pain stimulus, which is thought to be due to intrauterine-induced alteration of the hypothalamus-pituitary-adrenal axis (Schäffer et al., 2009). This phenomenon may also occur in vLBW piglets and explain the lower HCC levels in the hair of vLBW piglets found in the current study. However, further research on stress and stress responses in LBW pigs is needed to test this hypothesis.

## Conclusion

In conclusion, our results do not corroborate findings in humans, suggesting that other mechanisms may be underlying the occurrence of (v)LBW in humans than in pigs. These results may therefore not provide information that can be used in the investigation of vLBW occurrence and its effects in humans. They raise, however, a whole new set of questions about which mechanisms may be causing and influencing vLBW and its effects in pigs. Since we found an increased cognitive performance and reduced stress levels in vLBW as compared to NBW animals, it can be speculated that the conditions that lead to vLBW in pigs may trigger beneficial compensatory mechanisms, which may either arise pre- or early postnatally. As piglets face strong early postnatal competition, especially in large litters where vLBW occurs the most, such compensatory mechanisms can improve survival chances in vLBW animals. Looking further into these mechanisms is necessary in order to elucidate why effects of LBW are different between pigs and humans, which in turn might generate knowledge that can benefit both piglet welfare and improve animal husbandry practices.

## Conflict of interest statement

The authors declare that the research was conducted in the absence of any commercial or financial relationships that could be construed as a potential conflict of interest.
